# Anti-Inflammatory Effect of *Emblica officinalis* in Rodent Models of Acute and Chronic Inflammation: Involvement of Possible Mechanisms

**DOI:** 10.1155/2014/178408

**Published:** 2014-08-21

**Authors:** Mahaveer Golechha, Vikas Sarangal, Shreesh Ojha, Jagriti Bhatia, Dharmveer S. Arya

**Affiliations:** ^1^Department of Pharmacology, All India Institute of Medical Sciences, New Delhi 110029, India; ^2^Public Health Foundation of India, New Delhi 110070, India; ^3^Department of Pharmacology and Therapeutics, Faculty of Medicine & Health Sciences, UAE University, P.O. Box 17666, Al Ain, UAE

## Abstract

*Emblica officinalis*, commonly known as amla in Ayurveda, is unarguably the most important medicinal plant for prevention and treatment of various ailments. The present study investigated the anti-inflammatory activity of hydroalcoholic extract of *Emblica officinalis* (HAEEO). Acute inflammation in rats was induced by the subplantar injection of carrageenan, histamine, serotonin, and prostaglandin E_2_ and chronic inflammation was induced by the cotton pellet granuloma. Intraperitoneal (i.p.) administration of HAEEO at all the tested doses (300, 500, and 700 mg/kg) significantly (*P* < 0.001) inhibited rat paw edema against all phlogistic agents and also reduced granuloma formation. However, at the dose of 700 mg/kg, HAEEO exhibited maximum anti-inflammatory activity in all experimental models, and the effects were comparable to that of the standard anti-inflammatory drugs. Additionally, in paw tissue the antioxidant activity of HAEEO was also measured and it was found that HAEEO significantly (*P* < 0.001) increased glutathione, superoxide dismutase, and catalase activity and subsequently reduced lipid peroxidation evidenced by reduced malondialdehyde. Taken all together, the results indicated that HAEEO possessed potent anti-inflammatory activity and it may hold therapeutic promise in the management of acute and chronic inflammatory conditions.

## 1. Introduction

Inflammation plays a major role in rheumatoid arthritis and osteoarthritis [1]. In clinics, the nonsteroidal anti-inflammatory drugs (NSAIDs) are commonly prescribed for pain relief in arthritic conditions. However, their continual use is associated with serious adverse effects like gastric mucosal damage, occult blood loss and elevation of serum hepatic transaminases, salt and water retention, and also exacerbation of asthma [2]. In order to circumvent these adverse effects associated with conventional NSAIDs, novel selective COX-2 inhibitors are in progress. However, the development of serious adverse reactions, like cardiovascular events with rofecoxib and Stevens-Johnson syndrome with valdecoxib, has compelled their withdrawal from use [3]. Additionally, the clinical uses of the remaining drugs in this class have been prescribed with caution and have consequently decreased [4].

In milieu of these observations patients as well as health care providers prefer to use alternative therapeutic agents as they are considered to be safe and effective in alleviating inflammation associated with arthritis. Several Indian medicinal plants were reported as an important source of new chemical moieties with potential therapeutic effects [5]. The studies on plants with substantiated folkloric use as anti-inflammatory agents are viewed as a productive and logical research strategy in the search for new anti-inflammatory drugs

*Emblica officinalis* Gaertn. (*Euphorbiaceae*) commonly known as amla grow in the tropical areas of South-East Asia. The fruit of the plant is one of the most important medicinal ingredients used in Ayurveda, Siddha, Unani, Arabic, Tibetan, and various other folk systems for the management of myriad chronic ailments [6]. Experimental studies have shown potent antioxidant, analgesic, antipyretic, adaptogenic, immunomodulatory, and antiulcerogenic activities of the fruit of* Emblica officinalis* [6–8].

The fruits are reported to contain thermostable vitamin C, minerals, amino acids, tannins, flavonoids, and other important phytochemicals which are believed to possess diverse pharmacological and biological effects [9]. Earlier studies have shown that the leaf extract possesses anti-inflammatory activities in the carrageenan and dextran-induced rat hind paw edema [10]. However, studies on the fruit extract which is the most used part of amla have never been performed. Therefore, the present study was carried out to evaluate the anti-inflammatory activity of the hydroalcoholic extract of the fruit of* Emblica officinalis* (HAEEO) in both acute and chronic models of inflammation in rats. Further, in order to understand the possible underlying mechanism, the effect of extract on the oxidative stress produced by carrageenan was also studied in the rat paw.

## 2. Methodology

### 2.1. Plant Extract

The standardized lyophilized hydroalcoholic extract of the fruit of* Emblica officinalis* (HAEEO) was procured from Sanat Products Limited, India (A WHO-GMP and ISO 9001 Accredited Herbal Extract Manufacturer Company). The voucher specimen of lyophilized extract of the fruits of* Emblica officinalis *(number EO 0114) was deposited at Department of Pharmacology, All India Institute of Medical Sciences, New Delhi, India. The phytochemical analysis was done by using HPLC (Waters, Milford Massachusetts, USA). The extract obtained was of the highest purity with 28.26% w/w of hydrolysable tannins emblicanin A and emblicanin B on dried weight basis.

### 2.2. Drugs and Chemicals

Carrageenan, histamine, 5-hydroxytryptamine (serotonin), chlorpheniramine, cyproheptadine, prostaglandin E_2_ (PGE_2_), and bovine serum albumin were purchased from Sigma Chemicals, St. Louis, MO, USA. Indomethacin was procured from Cipla, India. All other chemicals and reagents were of analytical grade.

### 2.3. Experimental Animals

All experimental procedures described were reviewed and approved by the Institutional Animal Ethics Committee and care of animals was taken as per guidelines of CPCSEA, Ministry of Environment and Forest, Government of India. Wistar albino rats of either sex weighing 180–200 g were used for the study. The animals were procured from the central animal facility in All India Institute of Medical Sciences, New Delhi. The rats were group-housed in polypropylene cages with no more than four animals per cage. They were maintained under standard laboratory conditions with natural dark-light cycle and were allowed free access to standard pellet diet (Golden Feeds, India) and tap water* ad libitum*. All the experiments were carried out using five groups, each containing 6 animals (Groups I–V) except carrageenan-induced paw edema where Groups I–VI were used.

### 2.4. Determination of Anti-Inflammatory Activity of HAEEO on Acute Inflammation

#### 2.4.1. Carrageenan-Induced Hind Paw Edema in Rats

Acute inflammation was produced by injecting 0.1 mL of carrageenan (1% in saline) locally into the plantar aponeurosis of the right hind paw of the rats [11, 12]. Group I served as normal control, where no inflammation was induced. This group was used for evaluation of biochemical parameters. Groups II and III received vehicle (saline 1 mL/kg, i.p.) and standard drug indomethacin (10 mg/kg, p.o.), respectively, and served as vehicle and positive controls. HAEEO (300, 500, and 700 mg/kg, i.p.) was administered to Groups IV, V, and VI, respectively. The HAEEO or vehicle was administered 30 min prior to injection of carrageenan and indomethacin was orally administered 1 h prior to the injection of carrageenan. The pedal volume up to the ankle joint was measured using a digital plethysmometer (Ugo Basile, 7140 Comerio, Varese, Italy) at 0 h (just before carrageenan injection) and then at 3 h. The different timing was chosen because of the different route of drug administration. The % inhibition of edema volume between treated and control groups was calculated as follows: % Inhibition = *V*_*c*_ − *V*_*t*_ × 100/*V*_*c*_, where *V*_*c*_ and *V*_*t*_ represent the mean increase in paw volume in control and treated groups, respectively.

#### 2.4.2. Autacoids-Induced Hind Paw Edema in Rats

This experiment was conducted according to the method described by Singh and Pandey [13]. The autacoids serotonin (1 mg/mL), histamine (1 mg/mL), and prostaglandin E_2_ (1 *μ*g/mL) were employed as phlogistic agents. The effect of HAEEO (300, 500, and 700 mg/kg, i.p.) and vehicle was tested individually against each autacoid. The anti-inflammatory effect of HAEEO was compared with that of standard drugs against each autacoid: phenylbutazone (PBZ, 100 mg/kg, p.o.) against prostaglandin E_2_, chlorpheniramine (CPM, 3 mg/kg, p.o.) against histamine, and cyproheptadine (CPH, 3 mg/kg, p.o.) against serotonin. Right hind paw edema was induced by the subplantar injection of 0.1 mL of different phlogistic agents in the respective groups. HAEEO was administered i.p. 30 min prior to inflammatory insult and standard reference drugs were administered p.o. 1 h prior to the inflammatory insult. The pedal volume was measured just before (0 h) and then at 3 h after the phlogistic challenge.

### 2.5. Determination of Anti-Inflammatory Activity of HAEEO on Chronic Inflammation

#### 2.5.1. Cotton Pellet-Induced Granuloma in Rats

The cotton pellet-induced granuloma in rats was studied according to the method of D'Arcy et al. [14]. The animals were divided into five groups with six animals in each group. The rats were anaesthetized and sterile cotton pellets weighing 10 ± 1 mg were implanted subcutaneously into both sides of the groin region of each rat. Group I served as control and received the vehicle. HAEEO in the doses of 300, 500, and 700 mg/kg, i.p. was administered to animals in groups II, III, and IV for seven consecutive days from the day of cotton pellet implantation. Group V received indomethacin (10 mg/kg, p.o.) for the same period. On day 8, the animals were anaesthetized and the pellets together with the attached granuloma tissue were carefully removed and freed from extraneous tissues. The wet pellets were weighed and then dried in an oven at 60°C for 24 h to a constant weight; after that the dried pellets were weighed again. Increment in the dry weight of the pellets was taken as a measure of granuloma formation.

### 2.6. Determination of Levels of Oxidative Stress Parameters

The biochemical markers of oxidative stress were determined in the carrageenan-induced rat paw edema model. Animals were euthanized 3 h after measurement of paw volume and the inflamed paw tissue was removed and processed for the estimation of oxidative stress. Paw tissue samples were thawed and homogenized with 10 times (w/v) ice-cold 0.1 M phosphate buffer (pH 7.4). Aliquots of homogenates from paw tissue were used to determine the malondialdehyde (MDA) [15] and glutathione [16]. The remaining homogenates were centrifuged at 7000 rpm for 30 min at 4°C temperature and the supernatant was used for estimation of superoxide dismutase (SOD) [17], catalase [18], and protein [19].

### 2.7. Statistical Analysis

Data were expressed as mean ± S.E.M. Statistical differences between the treatment and the respective control groups were evaluated by one-way ANOVA followed by Tukey-Kramer post hoc test. *P* < 0.05 was considered to be statistically significant.

## 3. Results

### 3.1. Carrageenan-Induced Hind Paw Edema in Rats

**Figure 1:**
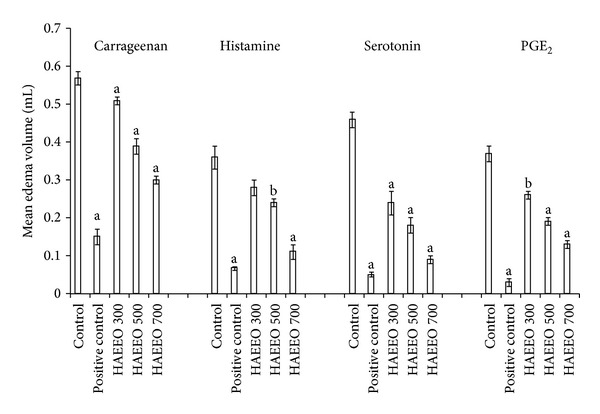
Effect of HAEEO on carrageenan- and autacoids-induced hind paw edema in rats. Each value represents the mean ± S.E.M. (*n* = 6). ^a^ *P* < 0.001 and ^b^ *P* < 0.01 compared to control. Positive control carrageenan (indomethacin 10 mg/kg), histamine (chlorpheniramine 3 mg/kg), serotonin (cyproheptadine 3 mg/kg), and PGE_2_ (phenylbutazone 100 mg/kg). HAEEO: hydroalcoholic extract of* Emblica officinalis*.

The mean increase in paw edema volume was 1.0 ± 0.02 mL in the vehicle-treated control rats. All the three doses of HAEEO (300, 500, and 700 mg/kg, i.p.) produced a dose-dependent significant (*P* < 0.001) reduction in the mean paw edema volume (Figure 1). The percentage inhibition in paw edema volume as compared to the vehicle treated group was 48.9, 60.2, and 70.0% for HAEEO, respectively. The standard drug, indomethacin (10 mg/kg, p.o.), exhibited maximum anti-inflammatory activity with 84.27% inhibition.

### 3.2. Effect of HAEEO on Changes in Tissue Levels of MDA, GSH, SOD, and Catalase

Carrageenan injection into the subplantar tissue of the rat paw decreased the tissue GSH, catalase, and SOD levels (Table 1). Both HAEEO and indomethacin produced a significant increase in the endogenous antioxidants in a dose dependent manner to maintain oxidative homeostasis. Carrageenan injection produced significant lipid peroxidation, as evidenced by a marked increase in the levels of MDA. Both HAEEO and indomethacin produced a significant decrease in the levels of MDA. HAEEO at 700 mg/kg dose most effectively stabilized the oxidative stress parameters.

### 3.3. Autacoid-Induced Hind Paw Edema in Rats

A dose-dependent effect of HAEEO on hind paw edema was observed. The 700 mg/kg dose of HAEEO was the most effective (Figure 1). It significantly (*P* < 0.001) inhibited hind paw edema induced by histamine (68.47%), serotonin (79.26%), and PGE_2_ (64.00%). Phenylbutazone (100 mg/kg, p.o.), chlorpheniramine (3 mg/kg, p.o.), and cyproheptadine (3 mg/kg, p.o.) also significantly (*P* < 0.001) inhibited hind paw edema induced by PGE_2_ (92.00%), histamine (82.06%), and serotonin (89.56%), respectively (Figure 1).

### 3.4. Cotton Pellet-Induced Granuloma

The study of HAEEO on proliferative phase of inflammation indicated that HAEEO (300, 500, and 700 mg/kg, i.p.) significantly (*P* < 0.001) and dose-dependently reduced the granuloma formation (Table 2). Indomethacin (10 mg/kg, p.o.) exhibited significant (*P* < 0.001) and maximum inhibition on granuloma formation.

## 4. Discussion and Conclusion

In the present study, it was observed that* Emblica officinalis* possessed potent anti-inflammatory activity both in acute and chronic rat models of inflammation. Inflammation is part of the host defense system and is triggered by a variety of noxious stimuli. It involves a complex interplay between cell-cell, cell-mediator, and tissue interactions [20]. Carrageenan-induced rat paw edema model is a well-established model for evaluating anti-inflammatory drugs [21]. The edema and inflammation induced by carrageenan are a biphasic event. In the initial 1 h after carrageenan administration, the edema and inflammation are mediated by histamine and serotonin. Later, the increased vascular permeability is maintained by the release of kinins up to about 2.30 h. Thereafter from 2.30 h to 6 h, inflammation is mediated by prostaglandins and is also associated with migration of leucocytes into the inflamed site [22].

Carrageenan-induced paw edema model in rats is known to be sensitive to cyclooxygenase (COX) inhibitors and has been used to investigate the effect of nonsteroidal anti-inflammatory agents [23]. The result of the present study indicated that HAEEO afforded protection against the carrageenan-induced acute inflammation in dose dependent manner. HAEEO at a dose of 700 mg/kg exhibited significant anti-inflammatory activity with 70.0% inhibition of paw edema and was comparable to the indomethacin group. In autacoid-induced models of inflammations (against serotonin, histamine, and PGE_2_), HAEEO produced significant inhibitory activity. The present study exhibited HAEEO's anti-inflammatory action by means of inhibiting the synthesis, release, or action of inflammatory mediators like histamine, serotonin, and prostaglandins that are involved in inflammation. In earlier study on the anti-inflammatory activity of leaf extracts of* Emblica officinalis* in carrageenan- and dextran-induced rat paw edema models, it was reported that the extracts did not inhibit the synthesis of the lipid mediators LTB_4_, TXB_2_, or PAF [24]. Therefore, it is quite possible that a composite effect may have been responsible for the observed protection against autacoids-induced inflammation.

The role of excess generation of nitric oxide (NO) in inflammatory response is well studied. Inflammation or tissue damage leads to induction of iNOS (inducible nitric oxide synthase); consequently large amounts of NO are generated at the site of inflammation [25]. NO reacts with superoxide anion to form peroxynitrite, an oxidizing molecule capable of eliciting lipid peroxidation. In lipid peroxidation there is oxidative deterioration of polyunsaturated lipids to form radical intermediates that causes cellular damage [26]. MDA is a major end product of this reaction and an index of lipid peroxidation that is measurable by estimating as thiobarbituric acid reactive substance (TBARS) [27]. The present study showed that both HAEEO (500 and 700 mg/kg) and indomethacin (10 mg/kg) decreased the levels of MDA.

The infiltrating inflammatory cells also generate reactive oxygen species (ROS) and free radicals. The most common ROS include the superoxide anion, hydroxyl radical, singlet oxygen, and hydrogen peroxide. The enzyme superoxide dismutase catalyzes the dismutation of superoxide into oxygen and hydrogen peroxide. The activity of SOD reduces during severe inflammation as well as in the presence of oxidative stress [28]. The large quantities of hydrogen peroxide generated are then taken care of by catalase and glutathione peroxidase (GPx) to water. Excessive production of lipid hydroperoxide may also lead to reduced activity of GPx in inflammatory conditions [29]. Besides the enzymatic antioxidants, the level of glutathione, a nonenzymatic reducing agent that traps free radicals and prevents oxidative damage, is also diminished in inflammatory conditions [30]. Both HAEEO (700 mg/kg) and indomethacin (10 mg/kg) maintained the oxidative homeostasis, and the levels of reduced glutathione and activities of catalase and SOD were comparable to the control animals.

Experimental studies have shown the potent antioxidant property of the fruit of* Emblica officinalis* [31]. Various phytochemical constituents of the plant such as emblicanins A and B, gallic acid, and ellagic acids have been identified as powerful free radical scavengers [9]. Moreover, other phytochemicals with NO scavenging properties like Geraniin, Corilagin, and Furosin have been reported in the* Emblica officinalis* fruit extract [32]. Recently, it has also been reported that the superoxide scavenging properties of* Emblica officinalis* extract approximate those of L-ascorbic acid, a well-established antioxidant [33].

In order to assess the efficacy of HAEEO against chronic inflammation, the cotton pellet granuloma model in rats was employed. HAEEO at all doses tested significantly (*P* < 0.001) reduced the granuloma formation. The maximum effect was observed at the dose of 700 mg/kg with 52.36% inhibition in granuloma formation as compared to the control group. Although the exact mechanism of anti-inflammatory activity of HAEEO on proliferative phase of inflammation in this model is not known, it may be hypothesized that both the antioxidant and the immunomodulatory properties of the plant may have been responsible for the protective action of the extract.* Emblica officinalis* extract has been reported to inhibit NF-*κ*B activation, a key transcription factor involved in chronic inflammatory response and ageing [34]. The inhibition of NF-*κ*B leads to reduction in the iNOS and COX-2 enzyme levels.

The main adverse effect of nonsteroidal anti-inflammatory drugs is their ability to produce gastric lesions [35]. Furthermore, Sairam et al. [36] demonstrated the ulcer protective potential of* Emblica officinalis* in different acute gastric ulcer models in rats induced by aspirin, ethanol, cold restraint stress, and pyloric ligation and healing effect in chronic gastric ulcers induced by acetic acid in rats. The antiulcerogenic activity of* Emblica officinalis* is definitely complementary to the good anti-inflammatory and antioxidant activity observed in the present study. Further, it has been shown that* Emblica officinalis *was well tolerated in mice even at the dose of 2.5 g/kg [37].

In conclusion, the present study clearly demonstrated that HAEEO possessed potent anti-inflammatory activity and also scientifically validated the traditional use of this plant for treating inflammatory disorders in the folk medicine. The advantages of HAEEO, namely, better and safer anti-inflammatory profile with potent antiulcerogenic activity, deserve further studies to establish the therapeutic value and elucidate the mechanism of action in the treatment of different inflammatory diseases.

## Figures and Tables

**Table 1:** Effect of HAEEO on oxidative stress parameters in carrageenan-induced paw edema in rats.

Treatment	GSH (*µ*g g^−1^ tissue)	MDA (nmol g^−1^ tissue)	SOD (Um g^−1^ protein)	Catalase (Um g^−1^ protein)
Normal control	32.91 ± 2.13	27.14 ± 2.96	40.54 ± 2.23	57.19 ± 2.48
Carrageenan control (vehicle treated)	13.33 ± 1.39^a^	88.45 ± 4.79^a^	15.19 ± 1.21^a^	14.48 ± 0.75^a^
Indomethacin (10 mg kg^−1^)	26.66 ± 1.66^b^	28.54 ± 6.85^b^	31.96 ± 1.08^b^	49.30 ± 1.86^b^
HAEEO (300 mg kg^−1^)	19.16 ± 1.53	63.18 ± 4.51^d^	21.18 ± 1.80	27.44 ± 1.66^b^
HAEEO (500 mg kg^−1^)	22.29 ± 2.80^d^	49.14 ± 5.83^b^	24.87 ± 0.98^d^	36.1 ± 0.83^b^
HAEEO (700 mg kg^−1^)	26.25 ± 2.18^c^	35.10 ± 2.78^b^	29 ± 1.66^b^	41.82 ± 1.41^b^

Values given are mean ± S.E.M. (*n* = 6). ^a^ *P* < 0.001 compared to normal control and ^b^ *P* < 0.001, ^c^ *P* < 0.01, and ^d^ *P* < 0.05 compared to carrageenan control. HAEEO: hydroalcoholic extract of *Emblica officinalis*; GSH: glutathione; MDA: malondialdehyde; SOD: superoxide dismutase.

**Table 2:** Effect of HAEEO on cotton pellet-induced granuloma in rats.

Group	Weight of cotton pellet granuloma (mg)	Protection percentage
Control (vehicle treated)	53.81 ± 1.94	—
Positive control (indomethacin 10 mg kg^−1^)	18.96 ± 2.18^a^	64.76
HAEEO (300 mg kg^−1^)	35.23 ± 1.48^a^	34.52
HAEEO (500 mg kg^−1^)	30.30 ± 0.94^a^	43.69
HAEEO (700 mg kg^−1^)	25.63 ± 1.29^a^	52.36

Each value represents the mean ± S.E.M. (*n* = 6). ^a^ *P* < 0.001 compared to control. HAEEO: hydroalcoholic extract of *Emblica officinalis.*
